# Chemically Induced Reprogramming of Somatic Cells to Pluripotent Stem Cells and Neural Cells

**DOI:** 10.3390/ijms17020226

**Published:** 2016-02-06

**Authors:** Dhruba Biswas, Peng Jiang

**Affiliations:** 1 Institute for Pediatric Regenerative Medicine, Shriners Hospitals for Children, Sacramento, CA 95817, USA; dhrubabiswas72@gmail.com; 2 Department of Developmental Neuroscience, Munroe-Meyer Institute, University of Nebraska Medical Center, Omaha, NE 68198, USA; 3 Holland Regenerative Medicine Program, University of Nebraska Medical Center, Omaha, NE 68198, USA

**Keywords:** reprogramming, small molecules, chemical reprogramming, induced pluripotent stem cells, neural stem cells, neurons

## Abstract

The ability to generate transplantable neural cells in a large quantity in the laboratory is a critical step in the field of developing stem cell regenerative medicine for neural repair. During the last few years, groundbreaking studies have shown that cell fate of adult somatic cells can be reprogrammed through lineage specific expression of transcription factors (TFs)-and defined culture conditions. This key concept has been used to identify a number of potent small molecules that could enhance the efficiency of reprogramming with TFs. Recently, a growing number of studies have shown that small molecules targeting specific epigenetic and signaling pathways can replace all of the reprogramming TFs. Here, we provide a detailed review of the studies reporting the generation of chemically induced pluripotent stem cells (ciPSCs), neural stem cells (ciNSCs), and neurons (ciN). We also discuss the main mechanisms of actions and the pathways that the small molecules regulate during chemical reprogramming.

## 1. Introduction

Adult neurogenesis in mammals including humans occurs mainly within two anatomical areas known as neurogenic sites: the forebrain subventricular zone (SVZ) [1] and the hippocampal dentate gyrus (subgranular zone, SGZ) [2]. In contrast, gliogenesis (formation of glial precursor cells) occurs continuously throughout the adult life in the CNS parenchyma, and these cells can differentiate into new oligodendrocytes and a low percentage of astrocytes [3,4]. In spite of this, the robust demand for neuron or glial cells replacement after brain injury is not met adequately through endogenous neurogenesis or gliogenesis, resulting in very limited self-repair. This has led to the notion that perhaps cell renewal occurring in adult neurogenic sites is primarily meant to be involved in tissue homeostasis, and hardly useful in response to external injury and neurodegenerative brain damage affecting the parenchyma. Nevertheless, these studies have demonstrated that newly formed neurons and glia can integrate into the adult CNS and contribute to brain functions. The current focus in regenerative medicine is therefore how to efficiently generate functional cell types, such as neural stem cells (NSCs), neuronal precursor cells, and glial cells including oligodendrocyte precursor cells (OPCs) and astroglia, that can efficiently integrate into the neural circuitry after transplantation [5,6,7].

During the past few years, several elegant studies have reported generation of iPSCs and neural cells using a new technology known as cell fate reprogramming, which allows for direct lineage conversion of mouse and human fibroblasts into iPSCs, neurons, and NSCs (albeit at a low efficiency). Conversion of one somatic cell type into a target cell of different cell lineage has been achieved, via ectopic expression of target specific transcription factors, or application of mRNAs, or exposure to small molecules. This reprogramming technology has the potential to evolve into a rapid, and direct approach for generating customized cell types, because the somatic cells (mainly skin derived fibroblasts) are easy to obtain and can generate autologous target cells with this technology. Mouse, rat, and human fibroblasts have shown to be directly converted into functional NSCs, neurons, astrocytes and OPCs by the ectopic expression of cell-fate-determining transcription factors or microRNAs. However, this process is technically challenging, because it shows low efficiency of induction, and the genomic integration of the viral vectors used for direct reprogramming have raised concerns about the potential for future applications of this approach. This had led to the initial hypothesis that an alternative strategy based on small molecules (chemical reprogramming) to induce cell-lineage reprogramming would be advantageous because such a strategy would be in theory non-immunogenic, lower cost, and easy to optimize. In addition, the application of small molecules is reversible and does not require cell permeabilization. Direct reprogramming of cell *in vivo* by small molecules could potentially lead to *in situ* regeneration therapeutic interventions. In this comprehensive review, we will summarize the recent advances in the field of chemical reprogramming for generation of iPSCs, NSCs and neurons from terminally differentiated somatic cells. In the last section, we will also discuss the mechanisms underlying the chemical-induced reprogramming in various reprogramming protocols.

## 2. Chemically Induced Reprogramming of Somatic Cells

### 2.1. Chemical-Induced Generation of iPSCs

Yamanaka and his colleagues, in their path breaking studies, demonstrated that ectopic expression of just four critical pluripotency genes under defined culture conditions reprogrammed a somatic cell into pluripotent stem cells, iPSCs [8]. Following Yamanaka’s concept, different groups were subsequently successful in showing that retro or lenti-viral vector mediated ectopic expression of lineage specific transcription factors can directly convert somatic cells to generate neurons [9,10], cardiomyocytes [6], oligodendrocytes [11,12], and hepatocytes [13]. This technology has two important advantages: (i) the direct reprogramming process bypasses the pluripotent state (potentially tumorigenic); and (ii) this process can generate autologous cells by reprogramming patients own somatic cells. However, the use of viral vectors and oncogenes for gene expression has generated valid concerns over the safe use of these cells in clinics. Recently, the field has shifted tangentially towards new chemical/small molecule-based reprogramming strategies and accelerated protocols. In pharmacology, a small molecule is defined as low molecular weight (<900 Daltons) organic bioactive compounds that may help regulate a biological process. Most drugs are small molecules. Huangfu *et al.* [14] were the first group to report the chemical assisted generation of ciPSC. They reported that by using the histone deacetylase (HDAC) inhibitor Valproic acid (VPA), they could eliminate the need for oncogenes *c-Myc* and *Klf4* (two of the four Yamanaka factors), and also found that the iPSC reprogramming efficiency was increased 100-fold over that of the four transcription factor method. Studies from Ding’s laboratory used the histone methyltransferase (HMT) inhibitor BIX-01294, to activate calcium channels in the plasma membrane, and improved the reprogramming efficiency using the four Yamanaka factors [15,16,17]. Lin *et al.*, 2009 [18] tested several inhibitors of transforming growth factor-β (TGFβ) receptor and MAPK/ERK kinase (MEK) on primary human fibroblasts (CRL2097 or BJ) that were transduced with retrovirus carrying genes encoding the four Yamanaka factors. They showed that a combination of seven small molecules improved the efficiency of iPSC generation from human fibroblasts by greater than 200 folds, within a week of treatment. Subsequently, several groups identified specific chemical combinations, which were sufficient to permit reprogramming from mouse embryonic and adult fibroblasts in the presence of a single transcription factor, Oct4, within three weeks, without the need for *Sox2*, *Klf4* and *c-Myc* [19,20,21]. The iPSCs developed using this protocol are similar to mouse ESCs in terms of expression of pluripotency genes, epigenetic state, and global gene expression profile (from RNA-seq data).

Hou *et al.* were the first to report all chemical generation of mouse iPSCs from mouse embryonic fibroblasts (MEFs) at a efficiency up to 0.2% using a combination of seven small-molecule compounds VC6TFZ: VPA, CHIR99021 (CHIR), 616452, Tranylcypromine, Forskolin (FSK), 2-methyl-5-hydroxytryptamine (2-Me-5HT), and D4476 [22]. This method also had a higher efficiency of induction compared to Yamanaka’s iPSC protocol (0.01%–0.1%). The chemically induced pluripotent stem cells (ciPSCs) exhibited similar global gene expression profiles as mouse ESCs. This study provided the proof of principle that by using small molecules, ectopic expression of master regulator genes is not necessary for cell fate reprogramming, thus showing the way for all-chemical reprogramming strategy with potential use in generating functionally desirable cell types for cell therapy. Although not reported to date, it is reasonable to speculate on the generation of human ciPSCs in the near future. Most small molecules that have been used so far to generate ciPSCs can be categorized as epigenetic modifiers, modifiers of cell signaling and apoptosis, wingless and integration site growth factor (WNT) signal modulators, moderators of cell senescence, or modulators of metabolism.

The fact that the reprogramming efficiency is less than 1%, even though the 100% of the donor cell population is exposed to small molecules, is very intriguing. There has been debate over two hypotheses, the “elite” and the “stochastic induction” of pluripotency, with respect to the methods by which these rare cells become iPSCs. In the stochastic model (the favorable model), the reprogramming process is initiated in most cells but only a few can achieve complete reprogramming. In the elite model, only a few cells are thought to be reprogramming competent. For a detailed discussion on these two models please refer to Yamanaka *et al.* [23]. It is proposed by Ladewig *et al.* that fibroblasts are reprogrammed to iPSCs via distinct intermediate stages, each with a variable recalcitrance to progression towards the next. One of these intermediate stages may be a hyper-proliferative phenotype, which is acquired stochastically with Yamanaka factor induction, but once obtained progresses deterministically to full pluripotency, and is parent to 99% of iPSC clones [24]. Very recently, Zhao *et al.* showed that the chemical reprogramming process of generating ciPSCs requires the early formation of extra-embryonic endoderm (XEN)-like cells that expressed Gata4, Gata6, Sox17, Sox7, Sall4 [25]. These cells were selectively primed to express Oct4 and establish pluripotency leading to a late transition from XEN-like cells to generate iPSCs, which fundamentally differed from the pathway of transcription factor-induced reprogramming. Manipulating the cell fate transition in a step-wise manner through the XEN-like state allowed them to screen and select small-molecule enhancers and establish a robust chemical reprogramming system with a yield up to 1000-fold greater than that of their previously reported protocol.

### 2.2. Chemical-Induced Generation of Neural Stem Cells

**Figure 1:**
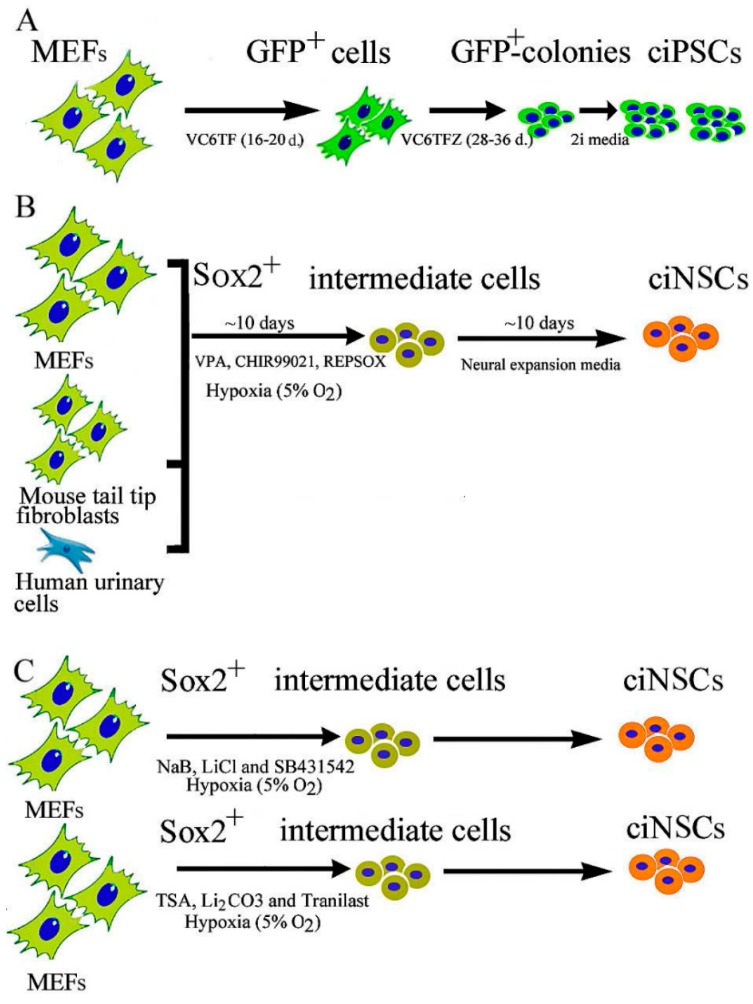
Chemically induced reprogramming of ciPSCs and ciNSCs. (**A**) A schematic diagram showing direct iPSC reprogramming from MEF using the small-molecule cocktail, VC6TF (VPA, CHIR99021, E-616542, Tranylcypromine, Forskolin), and VC6TFZ (VPA, CHIR99021, E-616542, Tranylcypromine, Forskolin and DZNep) followed by application of two MEK and GSK3-β inhibitors, also known as “2i”, to finalize chemical reprogramming. Using a doxycycline (DOX)-inducible GFP-Oct4 expression screening system, ectopic GFP-Oct4 expression was induced during the first round, followed by DOX withdrawal and small molecule treatment. Epigenetic modulators, particularly 3-deazaneplanocin A (DZNep), an S-adenosylhomocysteine hydrolase inhibitor, were later added along with MEK and GSK3-β inhibitors (2i), to achieve complete reprogramming; (**B**) The scheme of direct ciNSC reprogramming from MEFs, mouse tail fibroblasts, and human urinary cells using the small-molecule cocktail VCR (VPA, CHIR99021, and Repsox) and physiological hypoxia; (**C**) A schematic diagram showing direct ciNSC reprogramming from MEF using alternative cocktails NLS (sodium butyrate (NaB), Lithium chloride (LiCl) and SB431542) and TLT (Trichostatin A (TSA), Lithium chloride (Li2CO3) and Tranilast) and physiological hypoxia (5% O_2_).

Neural stem cells (NSCs) have now been recognized as a life-long source of neurons and glia in the adult brain. NSCs are self-renewing, multipotent cells that generate all the main CNS cell types, namely neurons, astrocytes and oligodendrocytes via exogenous stimuli from their extracellular environment. NSCs have demonstrated plasticity to generate, repair, and change nervous system function (excellent review by Gage and Temple) [26]. In 2012, Han *et al.* reported the direct reprogramming of mouse fibroblasts to induced neural stem cells (iNSCs) by ectopically expressing six transcription factors (Brn4/Pou3f4, Sox2, Klf4, c-Myc, plus E47/Tcf3) [27]. The iNSCs expressed NSC markers and resembled wild-type NSCs in their morphology, self-renewal, multi-potency, ability to form neurospheres, and gene expression profiles. Soon after, Ring *et al.* reported that ectopic expression of the single master transcription factor, Sox2 was sufficient to generate iNSCs from mouse and human fibroblasts [28]. Subsequently, in 2014, Cheng *et al.* were the first to report the generation of chemically induced neural precursor cells (ciNSCs) from MEFs, mouse tail-tip fibroblasts (TTFs) and human urinary cells (HUCs) by a cocktail of three small molecules “VCR”: VPA, an inhibitor of HDACs; C, CHIR99021, an inhibitor of GSK-3 kinases and R, Repsox, an inhibitor of TGF-β pathways, under a physiological hypoxic condition (5% O_2_), without introducing expression of exogenous genes [29]. This process was primarily accompanied by activation of endogenous Sox2 expression. They further showed that alternative small molecules cocktails “NLS” (combination of NaB, LiCl and SB431542) and “TLT” (combination of TSA, Li2CO3 and Tranilast) also could activate endogenous Sox2 expression in MEFs under the physiological hypoxic condition (Figure 1). These ciNSCs resembled mouse brain-derived NSCs in both cell properties and gene expression profiles, and differentiation to all the neural cell types both *in vitro* and *in vivo*. Thus, further development and optimization of this strategy to produce patient-specific NSCs may prove useful for treating neurological diseases including Alzheimer’s disease, Parkinson’s disease, as well as other conditions, such as pediatric genetic disorders.

### 2.3. Chemical-Induced Generation of Neurons

**Figure 2:**
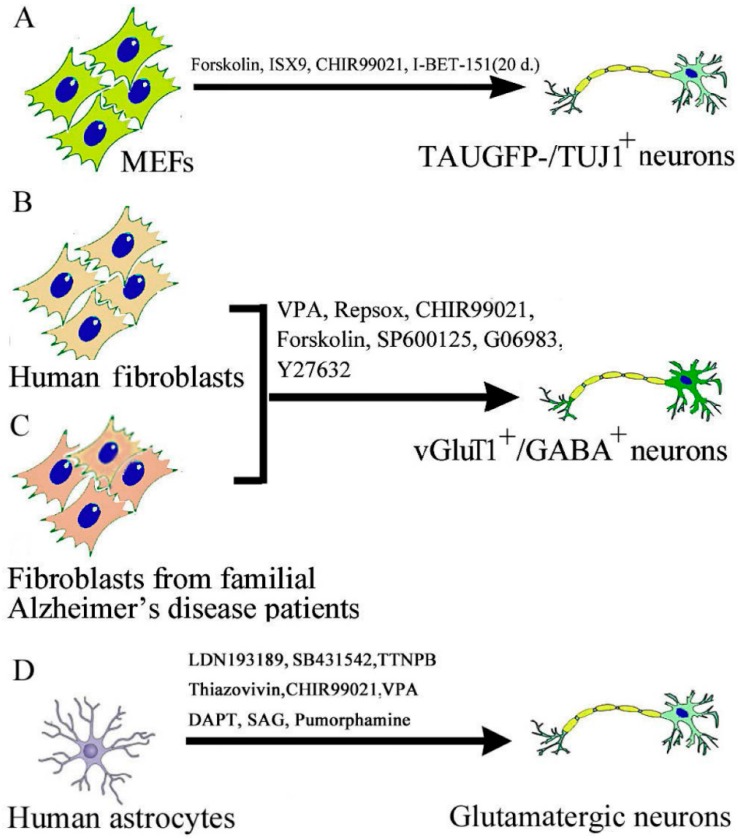
Chemically induced reprogramming of ciN. (**A**) A schematic diagram showing direct reprogramming to neurons from MEF using the small-molecule cocktail. Initially, four small-molecule cocktail “FICS” (Forskolin, ISX9, CHIR99021, and SB431542) efficiently induced TAUEGFP-/TUJ1-positive immature neurons within 21 days. Later addition of I-BET151 dramatically enhanced the reprogramming rate (with a 90% TUJ1-positive cell yield) and more mature ciN; (**B**) Human fibroblasts were directly converted into functional GABAergic and glutamatergic neuronal cells by a chemical cocktail VCRFSGY (valproic acid; CHIR99021; Repsox; SP600125 (JNK inhibitor), GO6983 (PKC inhibitor) and Y-27632 (ROCK inhibitor)), bypassing a neural progenitor stage; (**C**) This approach was further applied to generate hciNs from familial Alzheimer’s disease patients’ fibroblasts; (**D**) Human astrocytes were directly converted into functional GABAergic neurons by LDN193189, SB431542, TTNPB, Thiazovivin, CHIR99021, VPA, *N*-[*N*-(3,5-difluorophenacetyl)-l-alanyl]-*S*-phenylglycine *t*-butyl ester (DAPT), Smoothened agonist (SAG), and purmorphamine.

In 2015, two simultaneous articles from two research groups in China reported the successful generation of mouse and human neurons from dermal fibroblasts by an all-chemical approach (Figure 2). The study from Deng’s laboratory identified a cocktail of small molecules capable of establishing neuronal features in mouse fibroblasts efficiently and directly [30]. The reprogramming process was accompanied by disruption of the fibroblast-specific program, and activation of the endogenous expression of neuronal-specific genes. An initial chemical screening process was performed in this study to identify neuronal-fate-inducing small molecules. A set of three transcription factors Ascl1, Brn2, and Myt1l was previously shown to induce the generation of neurons from mouse fibroblasts, in which Ascl1 is the master gene for inducing neuronal fate, while Brn2 and Myt1l enhance the neuronal conversion as the supplementary factors [10]. Ascl1 alone (without Brn2 and Myt1l expression) also induces neurons with low efficiency [10]. Therefore, they first performed a chemical screening for small molecules promoting Ascl1-based conversion. After screening about 5000 small molecules, they found that “FICS”: Forskolin, ISX9, CHIR99021, and SB431542, each increased the number of TAU-EGFP-/TUJ1-positive neuronal cells induced by Ascl1 by more than two-fold (Figure 2A). In the presence of Ascl1, the combination of these four chemical enhancers further increased the efficiency of generating induced neurons (iNs) (>10-fold enhancement than Ascl1 infection alone without compounds. Thus, they identified a combination of “FICD” that robustly facilitate Ascl1-mediated induction of immature neurons from mouse fibroblasts. Further screening of 1500 molecules, led to identification of I-BET151, a compound that dramatically enhanced the reprogramming rate (with a 90% TUJ1-positive neuron yield).

Although the small molecules identified in these studies were not specific to the neural lineage, the pathways that they presumably have targeted have been reported to be involved in directed neural differentiation *in vitro* and even in neural development *in vivo*. CHIR99021 is reported to induce neuronal development from pluripotent stem cells as a GSK3 inhibitor [31], and it has been reported as a chemical booster that enhances transcription-factor-based neuronal conversion [32]. Forskolin is a diterpene adenylate cyclase activator and is commonly used to increase the level of cyclic AMP (cAMP) [33], and cAMP-responsive element binding (CREB), a downstream target of Forskolin, has been reported to regulate neuronal specification and promote axonal regeneration [33]. The results from the trans-differentiation studies suggest that extracellular developmental cues and development-related signaling pathways could be informative in developing small molecule combinations that facilitate cell lineage reprogramming.

The contemporary study from Pei’s laboratory for the first time directly converted human fibroblasts into neurons with a chemical cocktail consisting of seven small molecules [34]. They reported that “VCR” with the combination with four additional chemicals (Forskolin, SP600125, GO6983, and Y-27632) was able to induce 5% TUJ1-positive human chemically induced neurons (hciNs). Electrophysiological properties of “VCRFSGY” derived hciNs were similar to iPSC-derived neurons (Figure 2A). The hciNs showed high neuronal but low fibroblastic gene expression profiles. This approach was further successfully applied to generate hciNs from familial Alzheimer’s disease (FAD) patients (Figure 2B). The hciNs derived from FAD patient fibroblasts exhibited abnormal amyloid-beta production. The authors reported that the conversion process induced by their chemical strategy was accompanied by the downregulation of fibroblast-specific genes and the increased expression of endogenous neuronal transcriptional factors. It was consistent with the previous studies that the forced expression of certain neuronal TFs could convert mouse or human somatic cells into neuronal cells. Whereas VPA induces epigenetic modifications, inhibition of TGF-β and GSK-3 improves the neuronal conversion of stably transduced human fibroblasts by Ascl1 and Ngn2 [32]. Forskolin enables neurogenin 2 to convert human fibroblasts into cholinergic neurons [35]. SP600125 could facilitate the neural reprogramming of AHDF transduced with OCT4 alone [21]. Y-27632 assists in the maintenance of pluripotent stem cells and neuron survival [36]. Therefore, the chemical cocktail VCRFSGY erases fibroblast-specific gene expression of the initial cells, specifically upregulating neuronal gene expression and facilitating the neuronal conversion of HAFs. However, the precise regulatory mechanisms of this chemical cocktail are yet to be fully investigated.

Recently Zhang *et al.* reported that a cocktail of small molecules could reprogram human astrocytes into fully functional neurons in culture [37]. Specifically, sequential exposure to a combination of nine small molecules, including LDN193189, SB431542, TTNPB, Thiazovivin, CHIR99021, VPA, *N*-[*N*-(3,5-difluorophenacetyl)-l-alanyl]-*S*-phenylglycine *t*-butyl ester (DAPT), Smoothened agonist (SAG), and purmorphamine reprogramed astrocytes into functional neurons within 8–10 days (Figure 2C). The reprogramming process was mediated through epigenetic regulation and involves transcriptional activation of neuronal transcription factors NeuroD1 and Neurogenin2. After transplantation into mouse brain, the ciN survived for over one month and integrated into local neural circuits. Interestingly, mouse astrocytes could not be reprogrammed either *in vitro* or *in vivo* using this combination. These reprogramming studies may pave the way for future novel drug therapy for neural repair.

## 3. Mechanisms of Actions of Small Molecules in the Context of iPSC and Neural Cell Reprogramming

Small molecules are bioactive compounds that can modulate specific cellular pathways involved in cell signaling, transcriptional, metabolic, or epigenetic changes, all which are modulated during cellular reprogramming. If selective epigenetic modulation can be achieved with chemicals so that it can change the status of a transcription factor gene and its access to the promoter (inactive to active stage), then we will not require ectopic expression of that gene.

In general, three major classes of small molecule compounds that alter the epigenetic state have been studied in the context of reprogramming: modulators of (i) DNA methylation; (ii) histone acetylation; and (iii) histone methylation. Epigenetic modifications, specifically DNA hypermethylation, are believed to play an important role in the down-regulation and silencing of genes. Discussed below are the major small molecules in each category that are being used for reprogramming.

### 3.1. Modulators of DNA Methylation

The covalent addition of a methyl group to the carbon (at position 5) of the cytosine ring of DNA is known as DNA methylation. DNA methylation acts to inhibit transcriptional initiation by directly blocking transcription factor binding to the promoter, and also indirectly by promoting histone deacetylation resulting in chromatin condensation [38]). Silent genes are usually hypermethylated at their promoters, along with histone deacetylation, which then leads to repression of transcription and chromatin condensation. Active genes in contrast do not have promoter methylation, and usually exhibit histone lysine acetylation and histone methylation at H3K4, H3K36 and H3K79 marks. Recently, it has been suggested that reactivation of hypermethylated pluripotency genes is critical for complete reprogramming. DNA methyltransferase inhibitor 5-aza-cytidine (AZA) and the histone deacetylase (HDAC) inhibitor valproic acid (VPA) have shown to be able to increase reprogramming efficiency and even reduce the number of factors required for reprogramming. Two FDA-approved hypomethylating agents are 5-aza-cytidine and decitabine will be discussed below.

5-Aza-cytidine (5AZA) is a chemical derivative of the DNA nucleoside cytidine. It requires DNA incorporation for inhibition of DNA methyltransferase-1 (DNMT1) known to induce demethylation and reactivation of silenced genes. 5AZA was shown to facilitate the transition to full pluripotency of partially reprogrammed cell line, and to promote the number of embryonic stem cell-like colonies generated from fibroblasts [14]. It has also been confirmed that AZA treatment promotes reprogramming efficiency, and even reduces the number of factors required for reprogramming [39].

Decitabin is an FDA approved cancer treatment drug. A single treatment with decitabine, followed by five days in a defined neuronal transformation medium, followed by culturing in a neuronal maintenance medium converted human keratinocytes into cells that expressed mRNA for β3-tubulin and doublecortin after one week, and at the end of two weeks, expressed mRNA for NeuN, FOXP2, and NCAM1. After further culture, neurofilament-1, nestin, synapsin, FOXP2, and GluR1 proteins were detectable by immunostaining [40]. Treatment with decitabin could also induce human skin keratinocytes to express *Oct4* and the *OCT4* regulator mir-145 [41].

### 3.2. Modulators of Histone Methylation

In addition to DNA itself, the histone subunits of nucleosomes undergo cell type specific post-translational modifications that influence the degree of chromatin condensation and biologically modulate and stabilize transcriptional output by recruiting coactivators and repressors. Histone methyltransferases (HMTs), histone-lysine *N*-methyltransferase and histone-arginine *N*-methyltransferase, are enzymes that catalyze the transfer of one to three methyl groups from the cofactor *S*-Adenosyl methionine to lysine and arginine residues of histone proteins. The most widely reported inhibitor Bix-01294 is discussed below. Other inhibitors are Pargyline hydrochloride, 5′-Deoxy-5′-(methylthio) adenosine, *S*-(5′-Adenosyl)-l-homocysteine.

Bix-01294 is an inhibitor of G9a histone methyltransferase, an important regulator responsible for methylation of DNA and repression of transcription in pluripotent cells. It facilitates the reactivation of pluripotency genes in somatic cells, thus promoting reprogramming [16,17]. It has been reported that Bix-01294 can significantly improve the reprogramming efficiencies of *Oct4-Klf4*-expressing fetal neural progenitor cells into iPSCs [16,17].

### 3.3. Modulators of Histone Acetylation

HDACs play an important role in gene expression via alterations of chromatin structure. The chromatin complex consists of the DNA backbone, histone proteins, and non-histone proteins. The nucleosome is the basic repeating unit of chromatin. It is ~146 bp of DNA wrapped around each of the two copies of each of the four histones: H2A, H2B, H3, and H4. Gene expression is tightly regulated through the combined modifications of histones, including acetylation, phosphorylation, methylation, ubiquitination, and SUMOylation. HDACs are enzymes that remove acetyl groups from lysines of histones proteins and other regulatory and structural proteins and play critical roles in chromatin remodeling, and are involved in transcription regulation, cell-cycle progression, cell survival and differentiation. HDAC inhibitors are thought to mediate reprogramming through various processes, such as histone deacetylation, transcription factor or regulator deacetylation followed by chromatin remodeling [42]. Although only a very small number of protein-coding genes are affected by the action of HDAC inhibitors, almost half of the noncoding microRNAs are either upregulated or downregulated. Thus far, 18 HDACs have been identified. The most widely used HDAC inhibitor used for reprogramming is VPA.

VPA is an antiepileptic drug, in addition to its use in other neurological and psychiatric conditions. VPA enables reprogramming of primary human fibroblasts with only two factors, *Oct4* and *Sox2*, without the need for the oncogenes c-Myc or Klf4 [14] and improves the reprogramming efficiency of human neonatal foreskin fibroblast (HFF1) cells transduced with the four transcription factors two-fold [43]. The specific molecular mechanisms by which VPA may promote reprogramming through activating pluripotency genes at some intermediate stages is still not clearly understood [44]. For example, reprogramming with the four Yamanaka factors causes senescence in mouse fibroblasts, establishing a stress barrier for cell reprogramming. Administration of VPA protected the cells from reprogramming-induced senescent stress by increasing cell proliferation and inhibited apoptosis [45]. In addition, VPA also inhibited the G2/M phase blockage derived from the senescence stress [45]. Thus, one key role of VPA appears to be breaking the cell senescence barrier required for the induction of pluripotency [45]. Human iPSC reprogramming efficiency was improved by supplementing the histone deacethylase inhibitor, VPA, and Y-27632, an inhibitor of p160-Rho associated coiled-coil kinase (ROCK), after retroviral transduction [46].

Ppyrrole-imidazole polyamide (PIP) conjugated with suberoylanilide hydroxamic acid (SAHA) is a small molecule that can enhance the efficiency of reprogramming by directionally activating the transcription of *Oct3/4*, *Sox2*, *c-Myc* and *Klf4*. Mechanistically, the site specificity is conferred by PIP whereas SAHA functions as a deacetylase inhibitor [47,48].

### 3.4. Modulation of Cell Signaling

Pharmacological inhibition of four important kinases—Rho kinase, glycogen synthase kinase 3 (GSK3), TGF**β** receptors, and mitogen-activated protein kinase (MAPK) has been shown to enhance somatic cell reprogramming.

ROCK inhibitors: The Rho kinase (ROCK) isoforms, ROCK1 and ROCK2, are downstream targets of the small GTP-binding protein Rho. ROCKs signaling pathways mediate various important cellular functions such as proliferation, gene expression, motility, and cell shape, and presumably cross talk with other signaling pathways known to mediate reprogramming. ROCK inhibition enhances the survival of isolated reprogrammed iPSCs, thus improving the reprogramming efficiency [49]. Another rock inhibitor thiazovivin, can promote iPSC reprogramming when combined with SB431542 and PD0325901 by regulating cell-cell interactions [18].

GSK inhibitors: GSK3 is a serine/threonine kinase involved in the regulation of over 40 proteins in the cell [50]. It is an important part of the β-catenin/Wnt signaling pathway, which signals the cell to divide and proliferate. The Wnt pathway is an important pathway for stem-cell maintenance and proliferation. ESCs secrete Wnt ligands and this autocrine Wnt signaling is required to prevent their differentiation. It is also active in pathways that control cellular structure, growth, motility, and apoptosis. Inhibition of GSK3 has been shown to enhance reprogramming. Silva *et al.* showed that inhibition of MEK and GSK3 (by PD0325901and CHIR99021) promoted “pre-iPSC” into fully reprogrammed iPSC [51]. CHIR99021 has been identified as an enhancer that can significantly improve the reprogramming efficiency of *Oct3/4-Sox2-Klf4* (OSK) transduced MEFs, and allow reprogramming in the absence of *Sox2*, in combination with Parnate (an LSD1 inhibitor) [52]. A second GSK3 inhibitor, kenpaullone, can replace *Klf4* in reprogramming [53]. In canonical Wnt signaling, Inhibition of GSK3 with small molecules stabilizes β-catenin and allows it to translocate into the nucleus where it associates with TCF transcription factors to initiate transcription. Wnt signaling and GSK3 are important for stem-cell self-renewal and proliferation. Activation of the pathway with Wnt3a ligand stabilizes β-catenin and promotes reprogramming in MEFs with OSK transduction [54,55]. In addition, GSK3 has many protein targets and is involved in other signaling pathways including metabolic regulation. Further research is needed to define the mechanism through which CHIR99021 exerts its effects, which could then be exploited more specifically during reprogramming.

MEK inhibition: MAPK/ERK kinases (MEK) are a component of the Raf/MEK/ERK pathway, a highly conserved membrane receptor-to-nucleus signaling module. Extracellular mitogenic or differentiating signals bind to receptor tyrosine kinases through mediator proteins, and then activate the Ras GTPase and the downstream MAPK cascade in which successive downstream kinases are activated through phosphorylation. Extracellular signal regulated kinases (ERK1/2) are the last proteins in the MAPK/ERK signaling chain. They can regulate the activity of various genes by their ability to translocate to the nucleus and phosphorylate transcription factors. ERK activation is involved in a various cellular activities such as cell cycle arrest, cell proliferation, terminal differentiation, apoptosis, *etc.* For a detailed review, refer to Wang *et al.* [56]. When added during late stages of reprogramming, the MEK inhibitor PD0325901, combined with leukemia inhibitory factor (LIF) and GSK inhibition, stabilizes true iPSCs while inhibiting the growth of non-pluripotent cells and promoting the conversion of pre-iPSCs into a fully reprogrammed state [57]. Additionally, MEK inhibition prevents differentiation of iPSCs in culture, further enriching for the population of pluripotent cells. PD0325901 is a common supplement in stem-cell culture media for achieving pluripotency and during long-term passaging to prevent-non specific differentiation [57].

TGFβ signaling pathway inhibitors: The key proteins in the TGF**β** family, such as bone morphogenetic proteins (BMPs), have been shown to play a role in stem cell self-renewal and fate determination [58]. It has been shown that TGF**β** signaling plays important roles in reprogramming. Two inhibitors of TGFβ receptor 1 kinase (E-616451 and E-616452) had been identified as important reprogramming enhancers. During Yamanaka factor-mediated reprogramming, fibroblasts that originally belong to mesenchymal lineage undergo drastic morphological changes that result in iPSC generation, accompanied by the expression of E-cadherin, a marker for epithelial cells, (also highly expressed in hESCs) [18]. This mesenchymal to epithelial transition (MET) is a critical step in the generation of iPSCs. TGFβ pathway antagonists promote MET in fibroblasts, and substantially enhance generation of fully reprogrammed human iPSCs from fibroblasts through a significantly more efficient process. The widely reported TGFβ inhibitors, repsox, SB-431542, and A-83-01 are discussed below.

Repsox (E-616451) can replace *Sox2* to induce iPSCs from OKM-transduced MEFs in the presence of VPA [59]. In contrast, E-616452 can functionally replace transgenic *Sox2* in the absence of VPA and c-Myc, and thus it has been named as RepSox [59,60]. RepSox does not directly activate endogenous *Sox2*, but acts by triggering an endogenous switch in partially reprogrammed cells by Nanog expression, leading to fully reprogramming of fibroblasts [59,60].

SB-431542 is a non-specific inhibitor of TGFβ receptor 1 kinase, and can also replace Sox2 and Oct4 protein during reprogramming [61]. Furthermore, inhibition of TGFβ signaling early in reprogramming also alleviates the need for transgenic *c-Myc* expression [20,59]. The efficiency of generating iPSCs from human myoblasts is significantly improved by inhibitors of histone deacetylase (sodium butyrate) and TGFβ signaling (SB431542) [62].

A-83-01 is a TGF**β** inhibitor, and along with MEK and GSK-3 inhibitors, A-83-01 is critical for full reprogramming of rat iPSCs [63]. A-83-01, along with protein arginine methyltransferase inhibitor, AMI-5 enables reprogramming of MEFs transduced with Oct4 alone, and the derived iPSCs can give rise to live-born pups through tetraploid complementation.

## 4. Conclusions

Compared to virally mediated transcription factor gene induction, small molecules have a few distinct advantages in reprogramming: (i) they are easy to handle, store, titrate/optimize; (ii) their effects are rapid and reversible, and confined to different cellular compartments; and (iii) their effects can be optimized by varying their concentrations and combinations. Large throughput screening of small molecules to modulate specific targets or stem cell phenotypes have led to the generation and validation of useful compounds for enhancing cell-based therapy and/or facilitating the development of therapeutic drugs targeting endogenous stem and progenitor cells to treat degenerative diseases, cancer, and injuries. However, small molecules have their own disadvantages. Most of small-molecule-mediated actions are nonspecific, and often presents a challenge for using them and interpreting their effects. A specific small molecule may have multiple targets. Moreover, toxicity in humans may interfere with the clinical application of small molecules. Nevertheless, the potential of employing small molecules to advance the field of regenerative medicine should move forward with great strides. The recent advances in development of ciNSCs and ciNs are exciting. However, the mechanisms underlying the activity of these small molecules need to be fully elucidated. Future studies should focus towards more sophisticated pharmacological approaches to identify optimal concentrations, dose responses, exposure times, and synergistic effects in a systematic and high throughput assays.
